# Residual ridge height as a potential risk factor for membrane perforation during lateral-window sinus elevation surgery: a systematic review and meta-analysis

**DOI:** 10.1186/s12903-025-06902-6

**Published:** 2025-10-03

**Authors:** Mingfu Ye, Xiuwen Lin, Wenjun Liu, Javier Calatrava, Wenxia Huang, Hom-Lay Wang

**Affiliations:** 1 Implantology Department, Xiamen Stomatological Hospital, Xiamen Medical College, Xiamen, China; 2 Shanghai Key Lab of D&A for metal-Functional Materials, School of Materials Science& Engineering, Tongji University, Shanghai, China; 3 Department of Periodontics, Xiamen Stomatological Hospital, Xiamen Medical College, Xiamen, China; 4 Section of Graduate Periodontology, Faculty of Odontology, Complutense University of Madrid, Madrid, Spain; 5 Department of Periodontics, University of Michigan School of Dentistry, Ann Arbor, USA

**Keywords:** Schneiderian membrane, Sinus lift, Sinus augmentation, Complication, Bone height

## Abstract

**Purpose:**

Perforation of the sinus membrane is a common complication during maxillary sinus lift surgery. This systematic review aimed to evaluate if the residual ridge height (RRH) is a risk factor for membrane perforations during the lateral-window sinus lift surgery.

**Methods:**

PubMed, Embase, CENTRAL, Scopus, and Web of Science databases were searched for studies reporting the association between RRH and sinus membrane perforations during the lateral-window sinus lift surgery. The last date of the search was 17th June 2024.

**Results:**

Ten studies were eligible. 1601 patients undergoing 1809 sinus lift procedures were included in the studies. The overall sinus membrane perforation rate was 19.2%. The meta-analysis found that mean RRH was significantly lower in the perforation group than the non-perforation group (MD: -0.89 95% CI: -1.47, -0.31 I2 = 90%). The included studies classified Small RRH as a 3–4 mm ridge. Meta-analysis showed a tendency of increased risk of perforation with small RRH, but the difference was not statistically significant (OR: 2.47 95% CI: 0.49, 12.21 I2 = 88%). The exclusion of one outlier study showed statistically significant results.

**Conclusions:**

Small RRH seems to be a potential risk factor for sinus membrane perforation during lateral-window sinus lift surgery. More evidence is needed for improving the validity of the current results.

## Introduction

Bone resorption and pneumatization of the maxillary sinus are common problems encountered during the rehabilitation of posterior maxillary teeth with dental implants. Reduced residual ridge height (RRH) in this area is frequently decreased in long-term edentulous patients, significantly reducing the possibility of implant-supported tooth replacement [1]. Bone augmentation by elevation of the sinus membrane is still the most predictable and cost-effective method to overcome this problem. Furthermore, the scientific literature has shown that the success rates of dental implants placed in grafted maxillary bone are similar to those placed in the native bone [2, 3].

A maxillary sinus lift can be carried out mainly by two methods: the transalveolar or crestal method and the lateral window technique. There is no statistically significant difference in implant survival with either technique, and the method of choice is mostly based on the RRH [4]. The lateral window technique is usually employed when the RRH is < 5 mm, while the transalveolar technique can be used in cases with ≥ 5 mm of RRH[3]. The lateral window technique involves creating a window in the lateral wall of the maxillary sinus, followed by elevation of the delicate sinus membrane. Given the fact that the Schneiderian membrane is usually thinner than 1 mm, there is a high risk of membrane perforation, which can complicate the procedure [5]. Small perforations are commonly repaired with a suture or a collagen membrane, and the procedure can be completed successfully. Evidence suggests that there may not be a significant difference in implant survival rates with repaired sinus membrane. However, major perforations may not be reparable, which could lead to the abandonment of the surgery [6].

Several studies have described different risk factors for sinus membrane perforation. These can be classified as anatomical risk factors, like the presence of sinus septa, sinus pathology, sinus membrane thickness, lateral wall thickness, sinus contour, etc., or patient-related factors, like smoking [7]. Awareness of these risk factors can aid in treatment planning and anticipation of the complications so that they can be readily treated. Recently, several studies focusing on the lateral window technique [8–10] have also reported that RRH may be a risk factor for sinus membrane perforation; however, the evidence is still unclear. The lateral window technique is a highly technique sensitive procedure which is routinely used worldwide. Since sinus membrane perforation is the most common complication of the technique, identification of risk factors can be help clinicians anticipate perforation and take corrective measures. Thus, the aim of this systematic review and meta-analysis was to assess if RRH is indeed a risk factor for sinus membrane perforation during lateral window sinus lift surgery.

## Materials and methods

We designed and reported this review in accordance with the PRISMA [11] checklist. The review protocol was formulated and uploaded to PROSPERO (CRD42024556372) for registration prior to conducting the literature search. This is a Systematic Review and Meta-analysis, with Human Ethics and Consent to Participate declarations: not applicable.

### Inclusion criteria

The reviewers decided on the inclusion criteria before beginning the literature search. We aimed to include studies conducted on adult patients undergoing sinus lift surgery via the lateral window technique. The exposure variable was RRH, and the outcome variable was the perforation of the sinus membrane. Therefore, we included studies that classified the RRH as small or large on any cut-off and reported the risk of perforation in each group. We also included studies that reported RRH in perforation and non-perforation groups. There was no restriction on study design; both prospective and retrospective cohort studies, case-control studies, and secondary analyses of randomized controlled trials were eligible.

Based upon above criteria, the following PICO-S questions were generated:

- P (Population): Patients receiving implants in the posterior maxilla without enough RRH.
- I (Intervention): Lateral sinus lift with small RRH.
- C (Comparison): Lateral sinus lift with large RRH.
- O (Outcome): Sinus membrane perforation.
- S (Study Design): Clinical trials (prospective/retrospective) and RCTs.

Studies were excluded if the sinus lift was performed via the osteotome technique, were from the same investigation with overlapping or duplicate data, and whenever they were available only in non-English language or as abstract form. In case of duplicate presentation of data, the study with a maximum sample size was included.

### Search protocol

Two reviewers conducted a detailed electronic search for relevant studies published up to 17th June 2024. Both reviewers worked independently until the final step of study selection and resolved their differences by discussion. PubMed, Embase, CENTRAL, Scopus, and Web of Science databases were searched to look for studies based on the inclusion criteria. There was no restriction on the publication date of the article. The two reviewers devised a common search query by consensus and used it across databases. It consisted of relevant MeSH and free keywords combined with the Boolean operators ‘AND’ and ‘OR.’ The final search strategy was: ((((Schneiderian membrane) OR (sinus membrane) OR (sinus lift)) OR (sinus floor elevation) OR (sinus augmentation)) AND ((complication) OR (perforation))) AND ((residual bone height) OR (residual ridge height)). Search results of all databases were combined into a reference manager software. Search results were screened by the software for duplicates, which were automatically removed. The reviewers performed initial screening by reading the titles and abstracts of the articles. Selected studies underwent full-text analysis before final inclusion. Both reviewers also performed an additional search on Google Scholar to include gray literature and screened the bibliography of all selected studies for any other missed articles.

### Data management and study quality

A pre-defined data collection form was used to collect the following data: study author, year of publication, country, study design, sample size, number of procedures, mean age, gender, smokers, window preparation method, presence of sinus septa, number of missing teeth at the surgical site, mean RRH of the sample, perforation rate, and outcomes.

Two reviewers also performed the quality assessment of studies using the Newcastle-Ottawa Quality Assessment Scale (NOS) [12]. Every article was screened for selection bias, comparability of groups, and outcome assessment. The final score awarded to the study could vary from 0, indicating the highest risk of bias, to 9, meaning the lowest risk of bias. The reviewers cleared all disagreements by discussion.

### Statistical analysis

Two meta-analyses were conducted based on the available data. In the first one, the mean RRH between perforation and non-perforation groups was compared. Mean and standard deviation values of RRH were pooled to obtain the mean difference (MD) between the groups along with 95% confidence intervals (CI). In the second, study-specific cut-offs for small and large RRH were used, and the risk of perforation in the groups was calculated by calculating the odds ratio (OR) and 95% CI. All analyses were done on “Review Manager” (RevMan, version 5.3; Nordic Cochrane Centre - Cochrane Collaboration, Copenhagen, Denmark; 2014) in a random-effects model. The chi-square test and the I2 statistic indicated the heterogeneity between studies; I2 > 50% indicated substantial heterogeneity. Funnel plots and Egger’s test were used to examine publication bias. A leave-one-out analysis was conducted using the software itself to assess for any outliers. During the analysis, each study was excluded from the meta-analysis and the pooled effect size of the remaining studies was automatically calculated by the software. This enabled assessment if the results altered in significance on exclusion of any study.

## Results

**Fig. 1:**
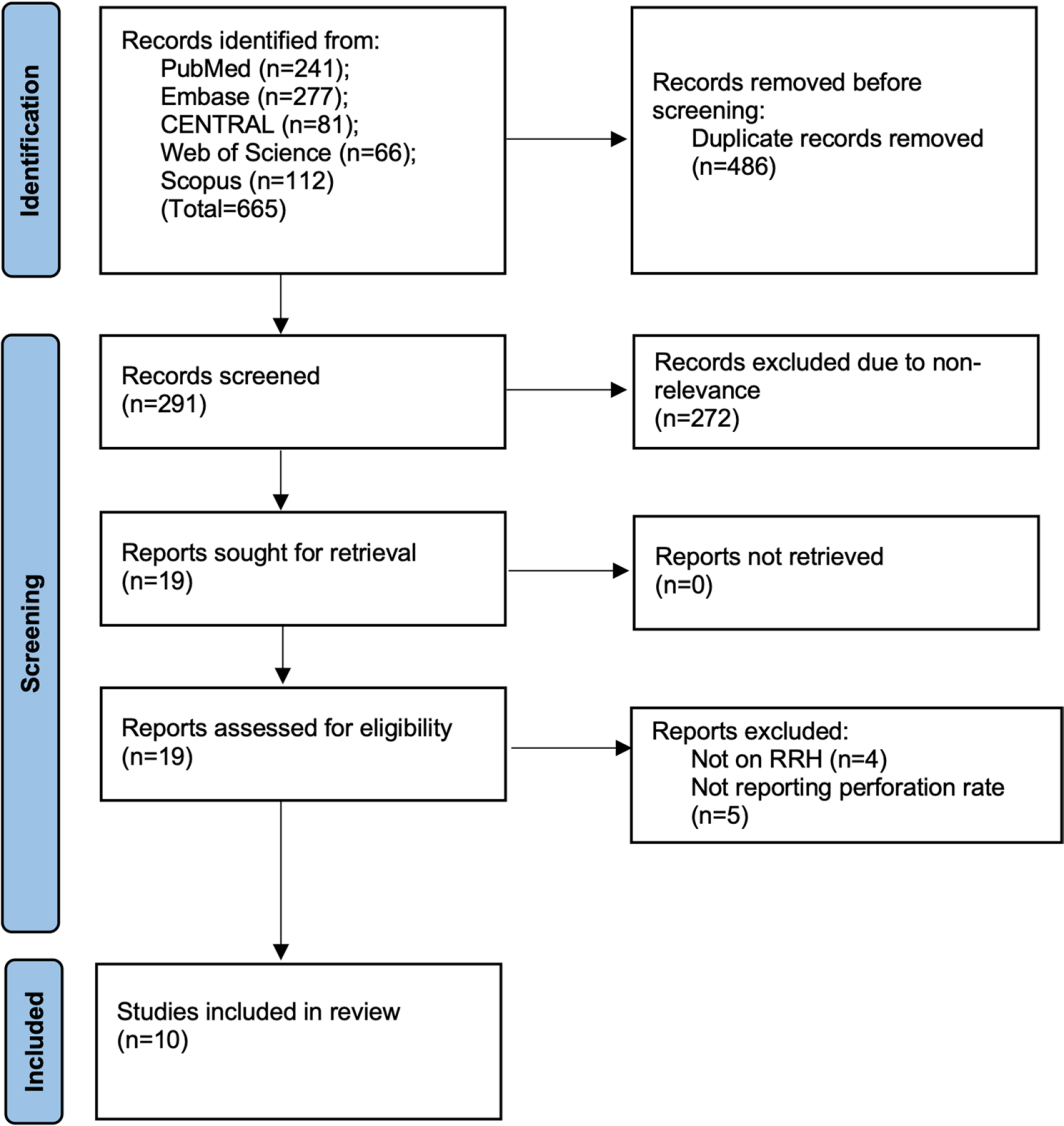
Study selection flow chart

Stepwise details of the study selection flowchart can be seen in Fig. 1. Initially, 665 studies were obtained after a detailed search of all databases. 291 unique articles were screened by abstract, and 19 were evaluated by their full texts. Finally, only ten met al.l the inclusion criteria and were included in this systematic review and meta-analysis [8–10, 13–19].

The included studies were all published after 2014 and originated from Austria, China, Iran, Korea, Switzerland, Turkey, and the USA (Table 1). Regarding their design, one study was prospective, while all others were retrospective studies. A total of 1601 patients were included in the studies and underwent 1809 sinus lift procedures. Patients’ mean ages ranged from 48.5 to 59 years, and the percentage of males ranged from 20.6 to 70.5%. Only one study did not report the percentage of smokers included. In the remaining studies, the proportion of smokers varied from 0 to 46.7%. For the creation of the lateral window, four studies used only rotary drills, while two studies used only piezosurgery, three used both, and one did not report data. Data on the presence of maxillary sinus septa at the surgical site was unavailable from two studies. In the remaining eight studies, the proportion of septa ranged from 0 to 47.8%.

**Table 1:** Details of included studies

Study	Location	Design	Sample size	Procedures	Mean age (years)	Males (%)	Smokers (%)	Drilling method	Septa (%)	Single missing tooth (%)	Mean RRH (mm)	Perforation rate (%)	NOS score
Nemati 2023	Iran	P	140	140	54.6	57.8	25.7	Bur	17.1	23.6	2.83 ± 0.94	15.7	7
Shao 2021	China	R	278	278	50.3	53.2	20.5	Bur/Piezo	12.2	15.7	NR	16.9	7
Krennmair 2021	Austria	R	355	434	55.9	49	42.8	Bur/Piezo	28.8	20.5	3.89 ± 1.51	23.8	7
Park 2019	Korea	R	63	65	59	63.5	39.6	Bur	NR	NR	NR	39	7
Tukel 2018	Turkey	R	120	120	53.5	58.3	46.7	Bur	47.8	NR	NR	18.3	7
Lum 2017	USA	R	167	167	48.5	56.9	8.9	Piezo	NR	NR	3.21 ± 2.02	28.1	7
Schwarz 2015	Austria	R	300	407	55.9	20.6	19	NR	27.1	NR	3.4 ± 0.8	8.6	7
Gurler 2015	Turkey	R	57	57	49.6	47.4	NR	Piezo	24.5	NR	4.04 ± 1.96	24.5	7
Von Arx 2014	Switzerland	R	77	77	57	37.7	24.7	Bur/Piezo	18.2	70.1	5.7 ± 1.97	27.2	7
Yimaz 2012	Turkey	R	44	64	51.4	70.5	0	Bur	0	NR	NR	17.2	7

*R* retrospective, *P* prospective, *NR* not reported, *NOS* Newcastle Ottawa scale

A total of 347 sinus membrane perforations were noted in the 1809 procedures conducted across studies, accounting for a pooled percentage of sinus membrane perforations of 19.2%. None of the studies conducted baseline matching comparisons of participants in the perforation and non-perforation groups. Hence, the reviewers awarded no points for comparability to any of the included studies. The final NOS score was seven for all studies.

**Fig. 2:**
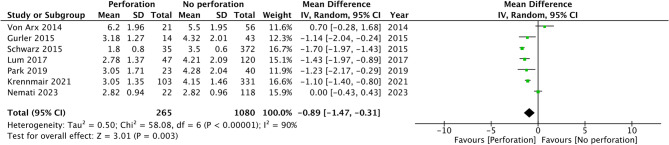
Meta-analysis of the difference in RRH height between perforation and non-perforation groups

**Fig. 3:**
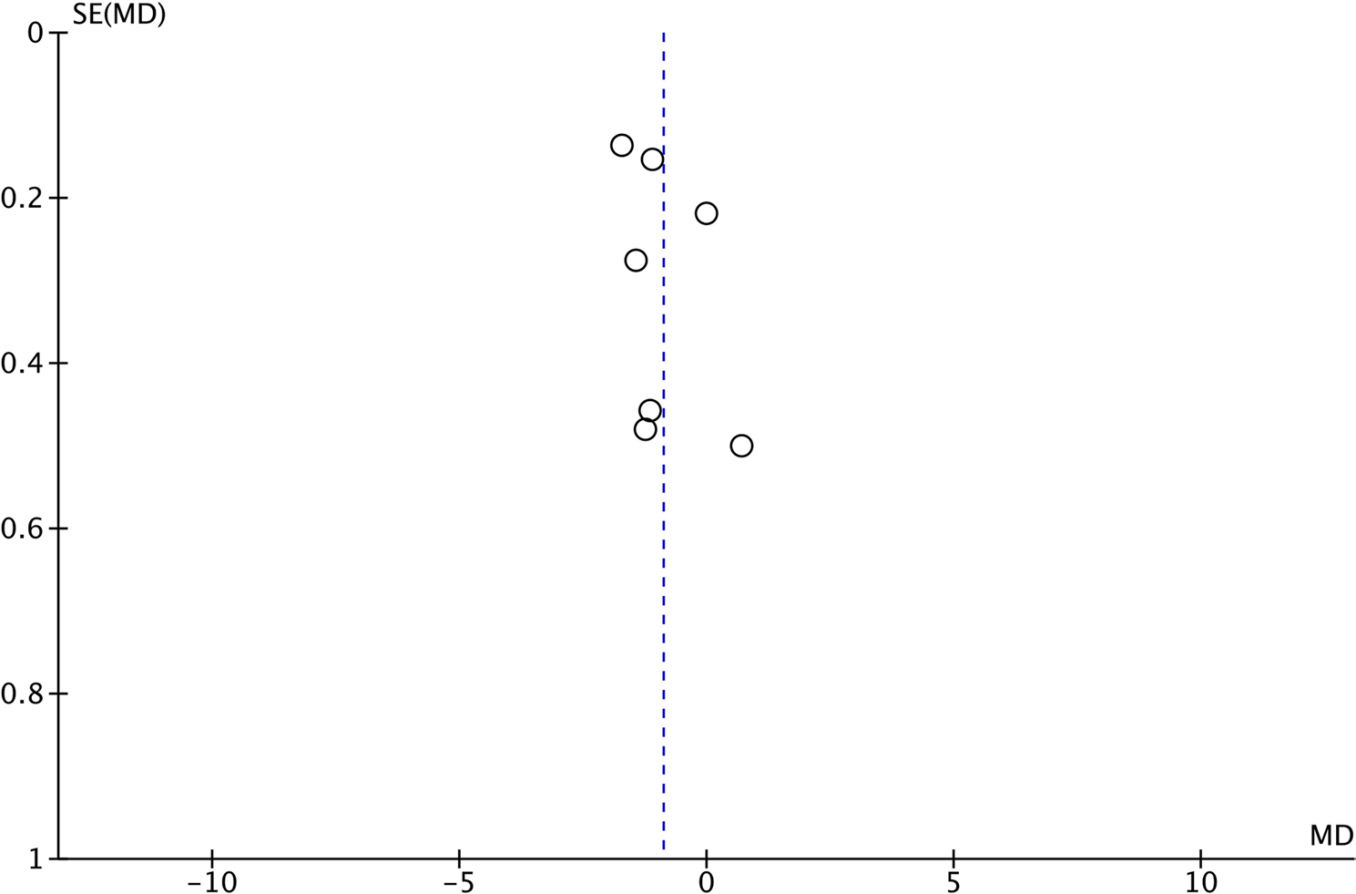
Funnel plot for publication bias

Seven studies reported the mean RRH in the perforation and non-perforation groups. Comparing data from 265 procedures with perforation and 1080 procedures without perforation, the meta-analysis found that mean RRH was significantly lower in the perforation group (MD: −0.89 95% CI: −1.47, −0.31 I2 = 90%) (Fig. 2). No publication bias was noted (Fig. 3). Egger’s test was not significant (*p* = 0.75). Furthermore, sensitivity analysis was conducted, excluding one study at a time, to check the significance of the results (Table 2). It can be noted that the MD varied from − 0.73 to −1.10 but the results remained statistically significant. However, there was no reduction in the inter-study heterogeneity with the exclusion of any study.

**Table 2:** Sensitivity analysis of mean difference in RRH between perforation and non-perforation groups

Excluded study	Mean difference [95% confidence intervals]	I^2^
Von Arx 2014	−1.10 [−1.65, −0.54]	89
Gurler 2015	−0.85 [−1.49, −0.21]	91
Schwarz 2015	−0.73 [−1.33, −0.12]	85
Lum 2017	−0.79 [−1.46, −0.12]	91
Park 2019	−0.84 [−1.47, −0.20]	91
Krennmair 2021	−0.83 [−1.61, −0.05]	91
Nemati 2023	−1.10 [−1.58, −0.61]	81

**Fig. 4:**
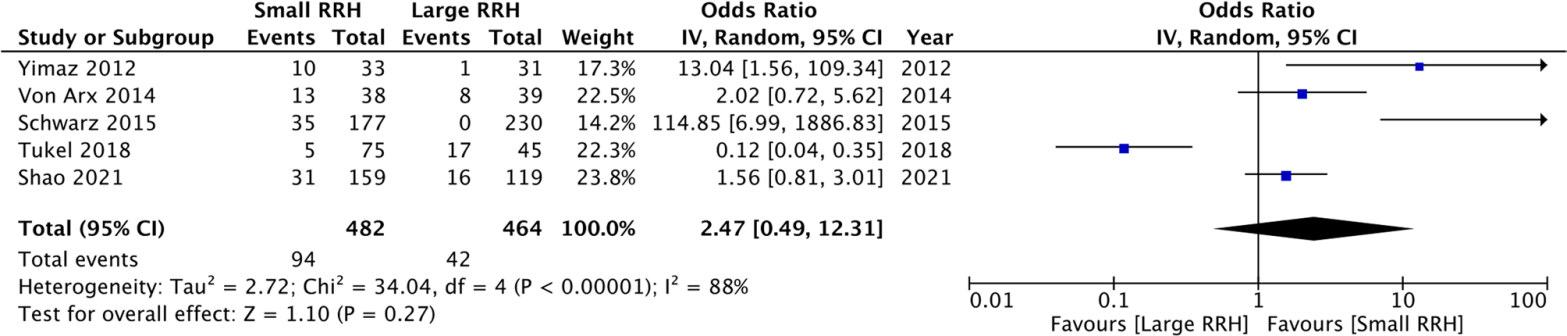
Meta-analysis of risk of perforation between small and large RRH

Five studies classified the RRH as small and large based on a cut-off point. Yilmaz et al. [9] and Schwarz et al. [18] used a cut-off of 3.5 mm, while Shao et al. [13] and von Arx et al. [10] used a cut-off of 4 mm. Lastly, Tukel et al. [15] used a cut-off of 3 mm. Overall, small RRH was therefore classified as a ridge of 3–4 mm. Meta-analysis showed that there was a tendency of increased risk of perforation with small RRH, but the difference was not statistically significant (OR: 2.47 95% CI: 0.49, 12.21 I2 = 88%) (Fig. 4). Egger’s test was not significant (*p* = 0.62). The results of the sensitivity analysis can be seen in Table 3. The study of Tukel et al. [15] was noted to be an outlier. The exclusion of this study showed that having a small RRH was a significant risk factor for sinus membrane perforation.

**Table 3:** Sensitivity analysis of risk of perforation between small and large RRH

Excluded study	Odds ratio [95% confidence intervals]	I^2^
Yimaz 2012	1.73 [0.30, 9.90]	90
Von Arx 2014	3.00 [0.32, 28.32]	88
Schwarz 2015	1.27 [0.28, 5.82]	87
Tukel 2018	4.68 [1.25, 17.55]	74
Shao 2021	3.43 [0.27, 43.12]	91

## Discussion

Sinus membrane perforation is one of the most common complications of sinus lift procedures, and its incidence has been reported to range from just 7% to up to 58% [20]. In addition to influencing the continuity or possible interruption of the surgical procedure, sinus perforations can also have important postoperative effects like acute or chronic sinus infections, bleeding, disruption of sinus physiological function, and loss of bone graft [21]. The impact of sinus membrane perforation on implant survival is, however, debatable. A long follow-up study including 379 implants has demonstrated that the presence of membrane perforations does not impact implant survival [22]. A recent systematic review and meta-analysis by Díaz-Olivares et al. [23], including seven articles and 1598 sinus lift surgeries, found that implants placed under perforated and repaired membranes had a survival rate of 97.68%, while those placed under non-perforated membranes had a survival rate of 98.88%, with no statistically significant difference. However, there have been contrasting reports in the literature. A detailed review of 85 studies has found an inverse relationship between perforation rates and implant survival, with every 10% increase in the incidence of perforation associated with a 55% chance of diminished implant survival [24]. In this context, the identification of risk factors for membrane perforation is of quite relevance so that the patient may be warned about possible risks and preparations be made for managing the complication.

This review presents the first aggregated evidence in the literature examining the exact role of RRH in increasing the risk of sinus membrane perforations. Given the available data, we conducted two types of data analysis. In the first part, we examined the mean RRH between cases of perforation and non-perforation, only to note a statistically significant reduced RRH in perforation cases. Detailed examination of the forest plot reveals that except for von Arx et al. [10] and Nemati et al. [8], all other studies reported significantly smaller RRH in perforation cases. Further adding to the evidence was the consistency of the results of the sensitivity analysis. However, the overall MD was small and was just 0.89 mm. Even though the results were statistically significant, the clinical significance of such a small MD may be questionable and needs to be further supported by future studies.

In the second meta-analysis, evidence was aggregated based on the classification of RRH as small or large. The meta-analysis showed that there was a tendency for increased risk of perforations with smaller RRH; nevertheless, the overall analysis did not demonstrate statistical significance. Examination of the forest plot revealed that the study of Tukel et al. [15] was the only one reporting opposing results. The reason for such results is unclear and could be attributed to the limited sample size of the study. All other studies noted a trend of increased risk of perforations with smaller RRH. On sensitivity analysis, the exclusion of Tukel et al. [15] indicated that a smaller RRH was associated with a 4.68 times increased risk of perforations, which was statistically significant. The most plausible explanation for such increased risk is that cases with smaller RRH need greater elevation of the sinus membrane to place an implant of the appropriate size. Since the tearing resistance of the membrane is limited, there could be an increased risk of perforations with smaller RRH [13].

This review has some limitations that should be acknowledged. High inter-study heterogeneity was noted in both of our meta-analyses. Since the included studies were conducted on different populations, with differing surgical protocols and varying surgeon experience, some heterogeneity was expected. However, there were important variations in patient-related and surgery-related factors that need to be considered while interpreting the current evidence. Firstly, the groups analyzed in the included studies were not matched for baseline variables and other risk factors for sinus membrane perforation. Most studies did not segregate data on such risk factors between the perforation and non-perforation groups or small RRH and large RRH groups. Also, variations in the proportion of smokers and sinus septa could be important confounders in the assessment of perforation rates, as they are both known risk factors for sinus membrane perforations [7, 8]. A recent meta-analysis of 11 studies has shown that sinus membrane thickness could also be a potential risk factor for membrane perforation [25]. However, most of the included studies failed to report and incorporate this factor into their analysis. It is plausible that the risk of perforation in the small RRH group may be due to higher prevalence of such risk factors which were not factored by the included studies. Only multivariate adjusted data or propensity scored matched data presented by future studies can overcome these limitations.

Furthermore, the risk of membrane perforation is highest during the preparation of the lateral window, and hence, the choice of the window preparation instrument is another important variable. The included studies used both rotary and piezosurgical instruments without any segregation of data. Literature shows that perforation rates are reduced with the use of piezosurgical instruments [26]. Differences in the reporting of outcome data and failure to report baseline risk factor data of the study and control groups precluded a detailed subgroup analysis in our review. Another limitation is the small number of studies available in the literature, which restricted the number of studies in each meta-analysis. The limited data can affect the depth and generalizability of the conclusions and hamper clinical application. The predominance of retrospective data also may introduce selection bias and errors due to data entry. Moreover, sinus lift surgery is a precise procedure, and the risk of complications can depend on the surgeon’s experience, but there was no data segregation based on surgeon experience available in the included studies. Lastly, the included studies differed in the cut-off of RRH, so a standardized threshold for a short RRH could not be established as a reference. Future studies should use cut-offs of both 3 mm and 4 mm to analyze the risk of perforation with RRH.

## Conclusions

Residual ridge height (RRH) was significantly lower in the cases when sinus membrane perforations occurred. Thus, small RRH (< 3–4 mm) could be a risk factor for sinus membrane perforations during maxillary sinus lift surgery. Surgeons should be careful when dealing with cases of small RRH and prepare in advance for managing possible membrane perforations. Further studies are needed to improve the quality of evidence.

## Data Availability

This is a Meta-analysis. All data generated or analysed during this study are included in this published article.

## Abbreviations

RRH: Residual Ridge Height
